# Thyroid cancer risk in the Swedish AMORIS study: the role of inflammatory biomarkers in serum

**DOI:** 10.18632/oncotarget.22891

**Published:** 2017-12-04

**Authors:** Arunangshu Ghoshal, Hans Garmo, Rhonda Arthur, Paul Carroll, Lars Holmberg, Niklas Hammar, Ingmar Jungner, Håkan Malmström, Mats Lambe, Göran Walldius, Mieke Van Hemelrijck

**Affiliations:** ^1^ King’s College London, School of Cancer and Pharmaceutical Sciences, Translational Oncology and Urology Research, London, UK; ^2^ Regional Cancer Centre, Uppsala University, Uppsala, Sweden; ^3^ Unit of Epidemiology, Institute of Environmental Medicine, Karolinska Institutet, Stockholm, Sweden; ^4^ AstraZeneca R&D, Mölndal, Sweden; ^5^ Department of Medicine, Clinical Epidemiological Unit, Karolinska Institutet and CALAB Research, Stockholm, Sweden; ^6^ Department of Medical Epidemiology and Biostatistics, Karolinska Institutet, Stockholm, Sweden; ^7^ Unit of Cardiovascular Epidemiology, Institute of Environmental Medicine, Karolinska Institutet, Stockholm, Sweden; ^8^ Endocrinology Department, Guy’s and St Thomas’ NHS Foundation Trust, London, UK; ^9^ Department of Palliative Medicine, Tata Memorial Hospital, Mumbai, India; ^10^ Biostatistics, Research & Development, Swedish Orphan Biovitrum AB, Stockholm, Sweden

**Keywords:** albumin, AMORIS cohort, cox analysis, serum inflammatory markers, thyroid cancer

## Abstract

Chronic inflammation is one of the underlying risks associated with thyroid cancer. We ascertained the association between commonly measured serum biomarkers of inflammation and the risk of thyroid cancer in Swedish Apolipoprotein-related MORtality RISk (AMORIS) study. 226,212 subjects had baseline measurements of C-reactive protein, albumin and haptoglobin. Leukocytes were measured in a subgroup of 63,845 subjects. Associations between quartiles and dichotomized values of inflammatory markers and risk of thyroid cancer were analysed using multivariate Cox proportional hazard models. 202 individuals were diagnosed with thyroid cancer during a mean follow-up of 19.6 years. There was a positive association between lower albumin levels and risk of developing thyroid cancer [Hazard Ratio for albumin ≤ 40 g/L: 1.50 (95% Confidence Interval = 1.04–2.16)]. When stratified by a metabolic score, we observed similar association for albumin with higher HR among those with metabolic score ≥ 1, as compared to those with metabolic score of 0 [HR 1.98 (95% CI = 1.11-3.54) vs 1.17 (95% CI = 0.72–1.89)] (*P =* 0.19). Apart from albumin, none of the serum markers of inflammation studied showed a link with the risk of developing thyroid cancer–suggesting that the role of inflammation may be more complicated and requires assessment of more specialised measurements of inflammation.

## INTRODUCTION

Thyroid cancer is the most common endocrine malignancy [1]. There has been an increase in its incidence in the Nordic countries, especially in Sweden between 1970 and 2013 [2]. Reactive oxygen species and proinflammatory cytokines generated in chronic inflammation are thought to initiate or promote cancer, and can affect disease progression [3]. For thyroid cancer, histological studies have confirmed the infiltration of white blood cell components such as CD8+ and CD4+ regulatory T lymphocytes in cancer tissues [4]. Moreover, increased levels of positive acute phase proteins like C-reactive protein (CRP), haptoglobin, galectin-3, IL6, G-CSF, sICAM-1, high neutrophil-to-lymphocyte ratio (NLR) and decreased levels of negative acute phase proteins like albumin, pre-albumin, and transferrin have been found in sera of patients with thyroid cancers [5–8]. Epidemiological studies have also suggested a link between chronic inflammation and risk factors for developing a thyroid cancer such as radiation exposure during childhood or as an occupational hazard [9], downstream genetic mechanisms in familial thyroid cancer syndromes [10], Hashimoto’s Thyroiditis [11], obesity [12], Hepatitis C-related chronic hepatitis [13] and increased parity and lactation [14].

Nevertheless, there are only a few observational studies exploring the link between inflammation and the risk of developing a thyroid cancer using systemic inflammatory markers. A recent study based on the European Prospective Investigation into Cancer and Nutrition (EPIC) cohort showed that insulin-like growth factor-I concentrations may be positively associated with the risk of developing differentiated thyroid carcinoma [15]. In a case-control study with 114 cases of thyroid cancer and 333 matched controls from the Janus serum bank in Norway, a positive association between serum albumin levels and the risk of developing papillary carcinoma and follicular carcinoma of the thyroid was found [16].

This study aimed to further evaluate this association. We explored the risk of developing a thyroid cancer by assessing serum biomarkers that are commonly measured in clinical practice and are indicative of inflammation (CRP, albumin, haptoglobin and leukocytes) in a large Swedish cohort study [17].

## RESULTS

Characteristics of study participants are shown in Table 1. During a mean follow-up of 19.6 years, 202 subjects developed thyroid cancer.

**Table 1:** Descriptive statistics of the study population by thyroid cancer status

	All (*n =* 226212)		Subgroup with additional measurement of leukocytes (*n =* 63845)	
	Thyroid cancer (*n =* 202)	No thyroid cancer (*n =* 226010)	Thyroid cancer (*n =* 66)	No thyroid cancer (*n =* 63779)
**Mean age (yrs.) (SD)**	48.28 (13.93)	45.77 (13.93)	51.2 (16.10)	50.6 (16.10)
**Gender**				
Male	72 (35.64)	120409 (53.28)	17 (25.76)	27559 (43.21)
Female	130 (64.36)	105601 (46.72)	49 (74.28)	36220 (56.79)
**Parity**				
Nulliparous	22 (10.89)	32411 (14.34)	7 (10.61)	9135 (14.32)
1+	127 (62.87)	146996 (65.04)	40 (60.61)	34210 (53.64)
Missing	53 (26.24)	46603 (20.62)	19 (28.79)	20434 (32.04)
**Socio-economic status**				
White collar	86 (42.57)	109373 (48.39)	23 (34.85)	28275 (44.33)
Blue collar	95 (47.03)	97858 (43.30)	28 (42.42)	24999 (39.20)
Unemployed/missing	21 (10.40)	18779 (8.31)	15 (22.73)	10505 (16.47)
**Education**				
Low	65 (32.18)	58892 (26.06)	18 (27.27)	15081 (23.65)
Middle	92 (45.54)	97205 (43.01)	32 (48.48)	26081 (40.89)
High	40 (19.80)	61025 (27.00)	14 (21.21)	17966 (28.17)
Missing	5 (2.48)	8888 (3.93)	2 (3.03)	4651 (7.29)
**Charslon Comorbidity Index**				
0	188 (93.07)	208707 (92.34)	59 (89.39)	56540 (88.65)
1	5 (2.48)	9107 (4.03)	3 (4.55)	3500 (5.49)
2	9 (4.46)	6085 (2.69)	4 (6.06)	2677 (4.20)
3+	0 (0.0)	2111 (0.93)	0 (0.0)	1062 (1.67)
**Body mass index (kg/m**^2^**)**				
< 18.5	1 (0.49)	829 (0.37)	0 (0.0)	200 (0.31)
18.5–24.99	20 (9.90)	27419 (12.13)	4 (6.06)	3920 (6.15)
25–29.99	14 (6.93)	14909 (6.59)	1 (1.51)	1855 (2.91)
> = 30	2 (0.99)	3623 (1.60)	0 (0)	491 (0.77)
Missing	165 (81.68)	179230 (79.30)	61 (92.42)	57313 (89.86)
**CRP (mg/L)**				
Mean (SD)	506 (3.0)	5.57 (3.0)	5.35 (4.0)	5.59 (3.0)
< 10	163 (80.69)	186574 (82.55)	54 (81.82)	52485 (82.29)
> = 10	39 (19.31)	39436 (17.45)	12 (18.18)	11294 (17.71)
**Albumin (g/L)**				
Mean (SD)	42.30 (42.0)	42.73 (43.0)	42.74 (43.0)	42.45 (42.0)
< 40	37 (18.32)	26725 (11.82)	7 (10.61)	9356 (14.67)
> = 40	165 (81.68)	199285 (88.18)	59 (89.39)	54423 (85.33)
**Haptoglobin (g/L)**				
Mean (SD)	1.03 (1.0)	1.05 (1.0)	1.09 (1.10)	1.08 (1.0)
< 1.4	174 (86.14)	194747 (86.17)	53 (80.3)	53816 (84.38)
> = 1.4	28 (13.86)	31263 (13.83)	13 (19.7)	9963 (15.62)
Leukocyte (109/L)^*^				
Mean (SD)			6.31 (5.95)	6.69 (6.30)
< 10			63 (95.45)	59607 (93.46)
> = 10			3 (4.55)	4172 (4.55)
**Fasting status**				
Fasting	133 (65.84)	139561 (61.75)	35 (53.03)	37791 (59.25)
Not fasting	69 (34.16)	84441 (37.36)	31 (46.97)	24625 (38.61)
Missing	0 (0)	2008 (0.89)	0	1363 (2.14)
**Glucose (mmol/L)**				
Mean (SD)	4.9 (0.96)	4.9 (1.28)	4.89 (0.68)	5.07 (1.46)
< 6.11	187 (92.57)	210508 (93.14)	63 (95.45)	57502 (92.13)
> = 6.11	15 (7.43)	13494 (5.97)	3 (4.55)	4914 (7.87)
Missing	0 (0)	2008 (0.89)	0	1363 (2.14)
**Triglycerides (mmol/L)**				
Mean (SD)	1.5 (2.1)	1.31 (1.0)	1.26 (0.74)	1.34 (1.05)
< 1.71	162 (80.2)	177854 (78.69)	53 (80.3)	49362 (77.4)
> = 1.71	40 (19.8)	46244 (20.46)	13 (19.7)	13048 (20.46)
Missing	0 (0)	1912 (0.85)	0	1369 (2.15)
**Total cholesterol (mmol/L)**				
Mean (SD)	5.60 (1.2)	5.60 (1.1)	5.52 (0.99)	5.63 (1.16)
< 6.5	158 (78.22)	177049 (78.34)	52 (78.79)	48427 (75.93)
> = 6.5	44 (21.78)	47060 (20.82)	14 (21.21)	13991 (21.94)
Missing	0 (0.0)	1901 (0.84)	0	1361 (2.13)

^*^Only measured in subgroup.

Lower values of serum albumin were associated with the risk of developing a thyroid cancer [HR for albumin ≤ 40 g/L: 1.50 (95% CI = 1.04–2.16)] in a multivariate model (Table 2). Additional adjustment for serum levels of triglycerides, glucose, and total cholesterol weakened the association slightly: HR 1.41 (95% CI = 0.97–2.04). No associations were observed for CRP, haptoglobin, or leukocytes with the risk of developing a thyroid cancer.

**Table 2:** Hazard ratio (HR) for risk of thyroid cancer with 95% confidence intervals (CI) using Cox proportional hazards model

	Crude	Adjusted^1^	Adjusted^2^
	HR (95% CI)	HR (95% CI)	HR (95% CI)
**CRP (mg/L)**			
> = 10	1.06 (0.75–1.51)	1.02 (0.72–1.46)	1.00 (0.70–1.43)
**Albumin (g/L)**			
< = 40	1.88 (1.31–2.68)	1.50 (1.04–2.16)	1.41 (0.97–2.04)
**Haptoglobin (g/L)**			
> = 1.4	1.09 (0.74–1.64)	1.02 (0.69–1.53)	0.99 (0.67–1.49)
**Leukocytes (10**^**9**^/L)^*^			
> = 10	0.75 (0.23–2.38)	0.75 (0.23–2.39)	0.73 (0.23–2.32)

^1^adjusted for age, sex, education, Charslon Comorbidity Index

^2^adjusted for age, sex, education, Charslon Comorbidity Index, triglyceride (continuous), glucose (continuous), total cholesterol (continuous), fasting status

^*^In subgroup with additional measurement for leukocytes *n* = 63,845.

When stratified by a proxy variable for obesity (metabolic score), we observed a similar positive association between albumin and the risk of developing a thyroid cancer in both groups, with a slightly higher HR for those with a metabolic score ≥ 1 [HR 1.98 (95% CI = 1.11–3.54)]) vs those with a metabolic score of 0 (1.17 (95% CI = 0.72–1.89)]. However, there was no statistically significant interaction (*P* = 0.19) by metabolic score (Table 3). No effect modification by sex was observed (results not shown).

**Table 3:** Hazard ratio (HR) for risk of thyroid cancer with 95% confidence intervals (CI) using Cox proportional hazards model stratified by metabolic score

	Metabolic score = 0 HR (95% CI)	Metabolic score ≥ 1 HR (95% CI)	P_Interaction_
**CRP (mg/L)**			
> = 10	1.10 (0.72–1.69)	0.82 (0.43–1.56)	0.37
**Albumin (g/L)**			
< = 40	1.17 (0.72–1.89)	1.98 (1.11–3.54)	0.19
**Haptoglobin (g/L)**			
> = 1.4	0.94 (0.54–1.66)	1.08 (0.59–1.94)	0.73
**Leukocytes (10**^**9**^/L)^*^			
> = 10	0.42 (0.06–3.09)	1.13 (0.26–4.90)	0.45

^*^ In subgroup with 63,845 measurements

All models were adjusted for age, sex, education, Charslon Comorbidity Index, triglyceride (continuous), glucose (continuous), total cholesterol (continuous), and fasting status.

**Figure 1:**
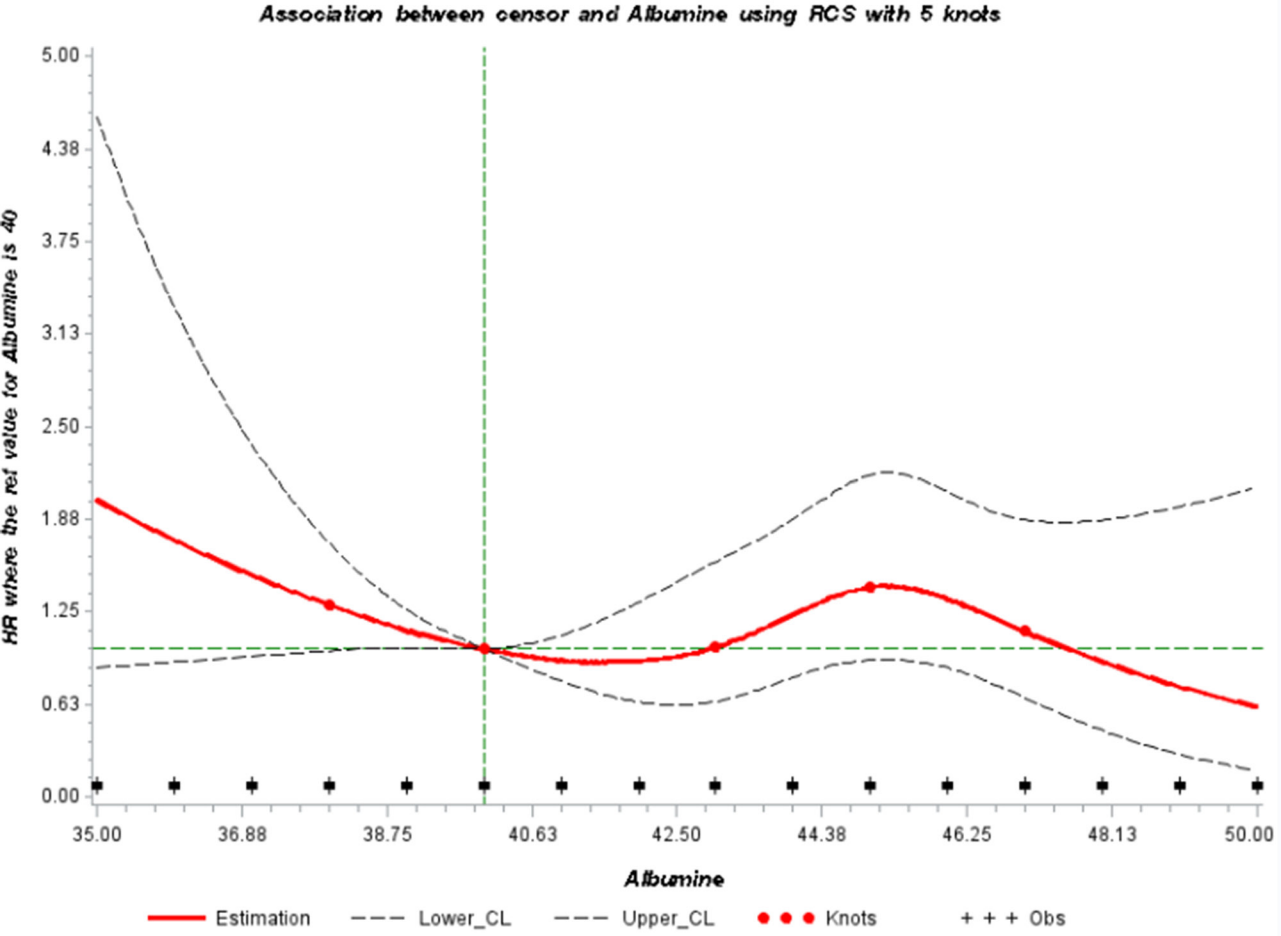
Adjusted dose-response association between serum levels of albumin (in g/L) and risk of thyroid cancer using restrictive cubic splines HR: Hazard ratio, RCS: restrictive cubic splines.

We further modelled the potential association between serum albumin and the risk of developing a thyroid cancer through a dose-response curve with restrictive cubic splines (Figure 1). The direction of the hazard ratios observed was consistent with shape of the curve.

A sensitivity analysis was done to assess reverse causation by excluding those with follow-up < 3 and < 5 years. It did not affect the above findings (results not shown).

## DISCUSSION

In the AMORIS study, we found evidence for an inverse association between serum albumin levels and the risk of developing a thyroid cancer. This inverse association became stronger when measured in a subgroup of subjects with serum triglycerides and total cholesterol above normal. No associations with the risk of developing a thyroid cancer were observed for CRP, haptoglobin, or leukocytes.

Molecular pathways linking inflammation and development of a thyroid cancer have been increasingly studied. For instance, increased expression of autotaxin, lysophosphatidate and inflammatory mediators has been found in malignant thyroid disease. It has been postulated that autotaxin is an integral component of tumorigenesis in thyroid follicular cells leading to tumour growth and promoting markers of more aggressive tumour phenotypes including proliferation, vascularity, metastasis, immune evasion, and treatment resistance [18, 19]. Moreover, inflammation is thought to play a role in papillary carcinoma of the thyroid, which is characterized by the presence of chemokine-guided macrophage and dendritic cell infiltration [20–22]. However, the majority of the studies investigating serum biomarkers and thyroid carcinogenesis have focused on progression and treatment response, with little focus on the risks of developing thyroid cancer [6, 8, 23].

The observed association between albumin and the risk of developing a thyroid cancer may support the well-established concept of low serum albumin as a marker of poor health status [24, 25]. Our results are different from a Norwegian case control study [16] and did not find a link between risk of developing a thyroid cancer and serum levels of CRP, haptoglobin and leukocytes, which is consistent with the few studies previously investigating this association [5, 6, 23].

This is to our knowledge the largest prospective study assessing common serum markers of inflammation with the risk of developing a thyroid cancer. The strength of this study lies in the prospective evaluation of exposures and complete follow-up of study participants through national health registers. All analyses were performed at CALAB using internationally accredited and calibrated methods [26]. The population in AMORIS is based on data from health check-ups in non-hospitalized persons and people referred for blood sampling, and is representative of the general working population of Stockholm [27–28].

It is a limitation that high-sensitive CRP was not available at the time measurements were conducted. Any CRP levels < 10 mg/L were unquantifiable, which may have resulted in an underestimation of the association with risk of developing a thyroid cancer. Information on BMI was not available for a large proportion in the current subset of AMORIS participants, so we used a metabolic score as an obesity proxy. Another limitation is the lack of data on histological variants of thyroid cancer [29], as well as other potential confounders such as smoking habits, diet and iodine consumption, exposure to ionizing radiation, thyroid endocrine abnormalities [30] or other serum biomarkers of inflammation such as IL-6 or IL-8. In addition, even though some studies have detected that overexpression of vascular endothelial growth factor C (VEGF-C) mRNA in thyroid cancer tissue has been associated with a high prevalence of lymph node metastasis, any such association could not be studied in the current AMORIS study due to lack of information on VEGF-C [31–35].

## MATERIALS AND METHODS

### Study population and data collection

The Swedish Apolipoprotein-related MORtality RISk data-base (AMORIS) includes blood samples from 812,073 Swedish men and women, ranging in age from < 20 to 80 years old and over undergoing occupational health screening or primary care. The cohort is based on a linkage between data from laboratory examinations performed in the Central Automation Laboratory (CALAB) in Sweden and information recorded in Swedish National Registers using a 10-digit personal identifier number, which is unique to every Swedish resident. AMORIS is a large prospective cohort with information on serum biomarkers, cancer diagnosis, co-morbidities, vital status, socioeconomic status, and emigration. This study complied with the Declaration of Helsinki and was approved by the Ethics Review Board of the Karolinska Institute.

We restricted our study population to individuals aged 20 years or older who did not have a previous diagnosis of cancer. Furthermore, all subjects were required to have baseline measurements of CRP, albumin, and haptoglobin available from the same health examination (*n* = 226, 212). Leukocytes were measured for a subgroup of the study population (*n* = 63, 845). The choice of biomarkers was guided by recommendations from similar studies done using data from the AMORIS study [36–38]. The outcome investigated in this study was development of a thyroid cancer (International Classification of Diseases, Revision 7 (1955) code 194) [39].

Serum CRP (mg/L), albumin (g/L), haptoglobin (g/L), leukocytes (10 × 109/L), triglycerides (mmol/L), total cholesterol (mmol/L) and glucose (mmol/L) were measured at baseline with standard laboratory methods as described elsewhere [40]. High sensitive CRP (hsCRP) was not available at any time in the period of blood sample collection (1985–1996), so CRP concentrations < 10 mg/L could not be measured precisely [26]. However, the cut-off point of 10 mg/L is widely accepted as the upper limit of the health-associated reference range and was therefore used in this study [41]. Levels of serum inflammatory markers were assessed as high or low based on their clinical cut-offs as used in CALAB: CRP 10 mg/L, haptoglobin 1.4 g/L and leukocytes 10 × 109/L. For albumin, a cut-off point of 40 g/L was used instead of 35 g/L due to the small number of participants with low albumin levels.

Serum glucose, total cholesterol, and triglycerides levels were dichotomised using clinical cut-offs in accordance with the American Diabetes Association and National Cholesterol Education Programme (NCEP) guidelines (cut-offs: 6.11, 6.50, and 1.71 mmol/L for glucose, total cholesterol, and triglycerides respectively) [42]. Obesity is linked to inflammation and development of a thyroid cancer [43–46], but limited information on body mass index (BMI) was available for this group of participants in AMORIS. Hence, we created a proxy variable (metabolic score) based on the sum of these three dichotomous variables indicating whether triglyceride, glucose, and total cholesterol were above their clinical cut-offs [37, 47]. In addition, the following baseline information was obtained from AMORIS: parity (nulliparous, 1+ children), educational level (low, intermediate, high), Charlson comorbidity Index (CCI; 0, 1, 2, ≥ 3), and fasting status (fasting, non-fasting, missing). Follow-up time was defined as the time from baseline measurements until date of cancer diagnosis, date of death, emigration, or end of study (31st December 2011), whichever came first.

### Data analyses

We estimated the risk of developing a thyroid cancer with multivariate Cox proportional hazards regression, comparing people with high to low levels of CRP, albumin, haptoglobin and leukocytes respectively. Cox regression models were adjusted for age, sex, education, and CCI, as well as triglycerides (continuous), glucose (continuous), total cholesterol (continuous), and fasting status. The assumption of proportional hazards was evaluated by adding time dependent covariates into the models as well as by assessment of the Schoenfeld Residuals.

We performed stratified analyses based on the values of the above-described metabolic score (0 vs ≥ 1) to evaluate effect modification by metabolic abnormalities. In addition, stratification by sex was performed.

Based on current evidence, the World Trade Center Program Administrator under Centers for Disease Control and Prevention (CDC) sets the minimum latency for developing a thyroid cancer as 2.5 years, which is well covered within our follow up time [48]. Nevertheless, we assessed reverse causality through two sensitivity analyses excluding those with follow-up time < 3 years and < 5 years, respectively.

For those biomarkers in which we observed an association based on the hazard ratios, we used the Restrictive Cubic Spline (RCS) function to graphically display the hazard ratios representing the dose-response association. We used knots located at the 5th, 25th, 75th and 95th percentiles as well as the medical reference value [49] in a multivariate Cox proportional hazards model as described above. This analysis was performed using the RCS_Reg SAS Macro created by Desquilbet and Mariotti [50].

All analyses were conducted with Statistical Analysis Systems (SAS) software release 9.4 (SAS Institute, Cary, NC) [51].

## CONCLUSIONS

The observation of an association between low levels of albumin and the risk of developing a thyroid cancer suggests the importance of inflammation as one of the mechanisms underlying carcinogenic development. The lack of an association with other commonly measured markers of inflammation indicates that the role of inflammation in the development of a thyroid cancer may be more complicated and would require more detailed data on markers of inflammation such as interleukins or gamma-delta T-cells. Future studies should entail temporality of associations between inflammation and development of thyroid cancers to further identify their clinical or public health importance.

## Abbreviations

AMORIS: Apolipoprotein-related MORtality RISk
CALAB: Central Automation Laboratory
CCI: Charlson Comorbidity Index
CDC: Centers for Disease Control and Prevention
CI: Confidence interval
CRP: C-reactive protein
EPIC: European Prospective Investigation into Cancer and Nutrition
g/L: gram per litre
G-CSF: Granulocyte-colony stimulating factor
HR: Hazard ratio
hsCRP: High sensitive CRP
IL6: Interleukin 6
IL8: Interleukin 8
mg/dl: milligram per decilitre
Mmol/L: millimoles per litre
NCEP: National Cholesterol Education Programme
NLR: neutrophil-to-lymphocyte ratio
RCS: restricted cubic splines
SAS: Statistical Analysis System
SD: Standard deviation
sICAM-1: serum Intercellular Adhesion Molecule 1
VEGF-C: vascular endothelial growth factor C
